# Identification of Novel Vaccine Candidates against *Campylobacter* through Reverse Vaccinology

**DOI:** 10.1155/2016/5715790

**Published:** 2016-06-16

**Authors:** Marine Meunier, Muriel Guyard-Nicodème, Edouard Hirchaud, Alberto Parra, Marianne Chemaly, Daniel Dory

**Affiliations:** ^1^Unit of Viral Genetics and Biosafety (GVB), French Agency for Food, Environmental and Occupational Health & Safety (ANSES), 22440 Ploufragan, France; ^2^Unit of Hygiene and Quality of Poultry and Pork Products (HQPAP), French Agency for Food, Environmental and Occupational Health & Safety (ANSES), 22440 Ploufragan, France; ^3^CZ Veterinaria, Porriño, 36400 Pontevedra, Spain

## Abstract

Campylobacteriosis is the most prevalent bacterial foodborne gastroenteritis affecting humans in the European Union. Human cases are mainly due to* Campylobacter jejuni* or* Campylobacter coli*, and contamination is associated with the handling and/or consumption of poultry meat. In fact, poultry constitutes the bacteria's main reservoir. A promising way of decreasing the incidence of campylobacteriosis in humans would be to decrease avian colonization. Poultry vaccination is of potential for this purpose. However, despite many studies, there is currently no vaccine available on the market to reduce the intestinal* Campylobacter* load in chickens. It is essential to identify and characterize new vaccine antigens. This study applied the reverse vaccinology approach to detect new vaccine candidates. The main criteria used to select immune proteins were localization, antigenicity, and number of B-epitopes. Fourteen proteins were identified as potential vaccine antigens.* In vitro* and* in vivo* experiments now need to be performed to validate the immune and protective power of these newly identified antigens.

## 1. Introduction

*Campylobacter* is the leading cause of human bacterial gastroenteritis in Europe [1]. It has been estimated that 9 million people are affected each year, costing around €2.4 billion.* C. jejuni* is responsible for approximately 90% of cases, and* C. coli* is responsible for 10%. Other species can cause human campylobacteriosis but are more rarely involved [2]. Most infections are not severe, leading to gastroenteritis symptoms, but they can cause extraintestinal manifestations such as reactive arthritis, Guillain-Barré syndrome (GBS), or inflammatory bowel disease (IBD) [3]. In some cases, infection can even lead to death. Human contaminations are mainly associated with handling and/or consuming poultry meat [1]. Domestic and wild birds constitute the bacteria's main reservoir, carrying up to 10^9^ CFU·g^−1^ of* Campylobacter* intestinally. In poultry flocks, natural colonization occurs in 2- to 3-week-old chicks by horizontal contamination from the environment [4], and birds remain infected until slaughter.

Decreasing avian colonization would appear to be an effective strategy for reducing the incidence of human campylobacteriosis. In 2013, Romero-Barrios et al. estimated that a reduction in* Campylobacter* cecal colonization from 2 to 3 log_10_⁡  units could lead to a 100% reduction in the risk of human disease [5]. Along with the implementation of biosecurity, hygiene, and nutritional measures in flocks, poultry vaccination is one way of reducing avian intestine colonization by* Campylobacter* [6]. Several vaccine prototypes have already been tested with variable results. These include whole-cell, subunit, or microorganism-vectored vaccines. Globally, whole-cell vaccines have not been efficient in decreasing* Campylobacter* intestinal loads despite the induction of a specific immune response [7–10]. Among subunit vaccines, flagellin—described as the immunodominant antigen of* Campylobacter*—has been tested and proved to be able to induce an immune response but this was not necessarily correlated with any decrease in chicken gut colonization [9, 11–13]. Furthermore, because of its weak homology across* Campylobacter* strains, flagellin-based vaccines do not induce cross-protection, making these vaccines inefficient in combatting all* C. jejuni* strains [14]. Other antigens such as CjaA [15]—a periplasmic protein—or CadF, FlpA, CmeC [16], and Dsp proteins [17] involved in* Campylobacter* adherence during colonization have also been trialed as subunit vaccines. In the same way, total outer membrane proteins [18] or fusion proteins [16] have also been tested. Another strategy is to deliver vaccine antigens by vectors such as attenuated bacterial strains.* Salmonella enterica* serovar Typhimurium [15, 19–21] and* Eimeria tenella* [22] have been evaluated as a vector for* C. jejuni* CjaA delivery. For example, in 2004, Wyszyńska et al. [19] indicated that chickens orally immunized with a virulent* Salmonella* strain carrying the* Campylobacter* CjaA gene develop a strong specific antibody response, and birds were protected from colonization after a homologous* C. jejuni* challenge. Recently, the same team was unable to confirm these results [21]. Other antigens were tested in the same way, including Omp18/CjaD, ACE393 [20], Dsp [17], Peb1A, GlnH, and ChuA [15]. Some of these experimental studies gave promising results, combining both the induction of a humoral immune response and a decrease in* Campylobacter* intestinal colonization in poultry, but experimentation has not yet been followed up. So, despite much research, no anti-*Campylobacter* vaccine aiming to reduce bacterial colonization in the poultry gut is yet available.

Identifying new potential vaccine antigens is one way of speeding up the development of new vaccines. Reverse vaccinology—a recent approach first described by Rappuoli in the early 2000s [23]—is used to predict antigens through the development of genomics and bioinformatic tools such as genome sequencing. This strategy is different from Hoppe et al. approach, where they identified novel immunodominant proteins by* in vitro* screening of mRNA of* C. jejuni* [24]. The following selection criteria are of particular importance for the reverse vaccinology approach. To be potentially good candidates, the selected proteins must be surface-exposed and able to be recognized by the immune system. Proteins with adhesin capacities are known to be involved in bacterial pathogenicity and invasion, so adhesins or adhesin-like proteins appear as good vaccine targets. The transmembrane helix number is also an important criterion. Indeed, it is difficult to purify proteins with more than one transmembrane helix, and it seems wise to exclude these proteins from the selection process [25]. Individual antigenicity and B-epitope density (the ratio between the number of B-epitopes and the protein length) need to be assessed as described by Oprea's study aimed at developing a vaccine against* S. aureus* endocarditis [26]. Although a few studies are describing innate intestinal inflammations and gut mucosa lesions upon* Campylobacter jejuni* infection (like in [27]), these bacteria are mostly described as a commensal organism for poultry [28]. In the avian intestinal tract, intensive* Campylobacter* multiplication occurs in the mucus layer of the epithelial cells. In this way, antigens need to induce a humoral immune response to neutralize and eliminate* Campylobacter* from the avian intestinal gut. The induction of a cytotoxic cellular response may not be a selection criterion since* Campylobacter* multiplication in intestinal epithelial cells of chickens was not clearly highlighted [28]. Anyway, bioinformatic tools aiming at predicting T epitopes for avian vaccines are still poorly developed, limiting the reverse vaccinology analysis in this goal. Finally, to provide cross-protection and avoid autoimmune response, it is essential that vaccine candidates are common to many pathogenic strains and do not mimic host proteins [25].

Our research identifies new potential vaccine antigens against* Campylobacter* using the reverse vaccinology strategy to develop an avian vaccine which could impact the incidence of human campylobacteriosis.

## 2. Material and Methods

### 2.1. Bacterial Strain

The highly virulent* Campylobacter jejuni* subsp.* jejuni* 81-176 strain was chosen for this* in silico* analysis. Its genome is available on the NCBI website under accession number CP000538.1 and listed in the Vaxign program used below.

### 2.2. OMP and Extracellular Protein Preselection

Vaxign (http://www.violinet.org/vaxign/index.php) was used to shortlist proteins with potential as vaccine candidates due to their cellular localization, probability of having adhesin-like characteristics, and number of transmembrane helixes [25]. Vaxign is a web-based pipeline dedicated to vaccine design and integrating several bioinformatic programs. Subcellular localization is predicted using PSORTb2.0 [29]. The probability of adhesin characteristics is predicted by SPAAN software [30] and the transmembrane helix topology is predicted by HMMTOP [31] using a hidden Markov model.

*Campylobacter jejuni* subsp.* jejuni* 81-176 is available in the Vaxign database of over 350 listed genomes, along with nine other* Campylobacter* genomes. Extracellular and outer membrane proteins having an adhesin probability score > 0.51 and either 1 or 0 transmembrane helixes were preselected.

### 2.3. Protein Antigenicity

VaxiJen v2.0 (http://www.ddg-pharmfac.net/vaxijen/VaxiJen/VaxiJen.html) was used to predict protein antigenicity. This software uses the physicochemical properties of proteins to predict their antigenicity from FASTA-submitted amino acid sequences. This feature is characterized according to an antigenic score. Proteins with an antigenic score > 0.5 were selected as described by Doytchinova and Flower [32].

### 2.4. Epitope B Prediction

BCPreds software (http://ailab.ist.psu.edu/bcpred/) was used to identify B-cell epitopes in FASTA-submitted amino acid sequences. This program provides two methods based on different algorithms: the amino acid pair (AAP) antigenicity method [33] and the BCPreds method using string kernels [34]. These methods predict antigenic linear nonoverlapping 20-mer epitopes from the whole antigen. Each preselected protein was analyzed and B-cell epitopes with a score >0.8 were accepted (specificity > 80%). The selected epitopes were again submitted to VaxiJen software to check their individual antigenicity and those having an antigenic score >0.5 were selected. Furthermore, for each protein and each algorithm, the ratio of B-epitopes to protein length was calculated to assess B-epitope density.

### 2.5. BLAST

In order to assess conservation of the selected proteins in the different Campylobacter strains, tblastn analyses were performed for each amino acid sequence against both* C. jejuni* and* C. coli* whole genomes available on the NCBI site on the day of analysis (February 9, 2016): 93 for* C. jejuni* and nine for* C. coli*. The identity percentage was set to 80% and the minimum query coverage was set to 50%. The amount and percentage of sharing among the available genomes were determined. The proteins with a sharing percentage lower than 80% (i.e., about the value for the flagellin) were eliminated from the protein shortlist.

A blastp analysis was also performed to ensure that the host* Gallus gallus* does not express the selected proteins. The identity percentage was set to 50% and the minimum query coverage was set to 50%.

## 3. Results

**Figure 1:**
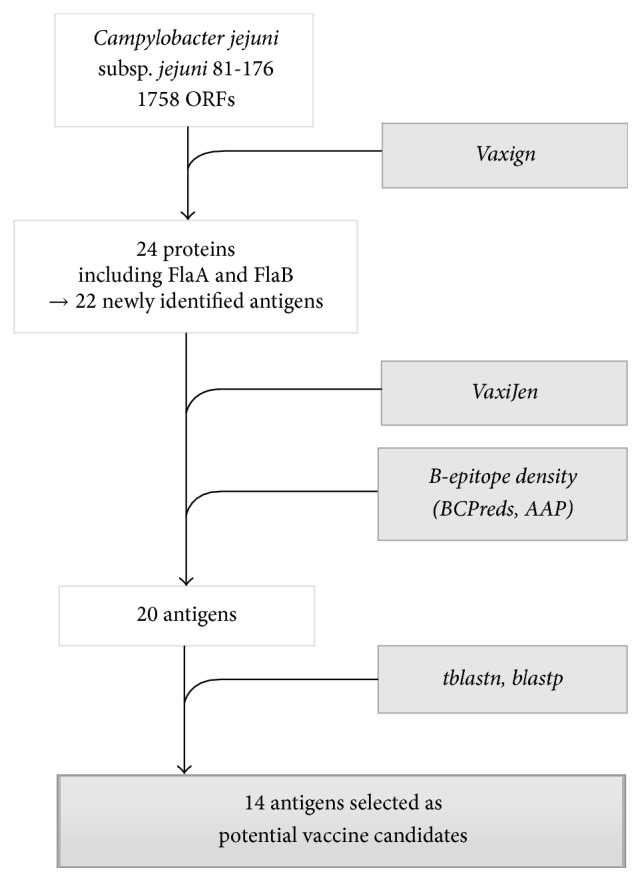
Summary of the reverse vaccinology protocol applied to* Campylobacter jejuni* for the selection of vaccine candidates.

The reverse vaccinology protocol applied here and results are summarized in Figure 1.

### 3.1. Protein Preselection

The Vaxign server was used to preselect vaccine candidates. Of the 1758 ORFs encoded by the* C. jejuni* 81-176 genome, only 24 were identified as potential vaccine antigens according to the applied criteria (localization, adhesion features, and number of transmembrane helixes) (Table 1). Of these 24 identified ORFs, we found the two known flagellins A and B, which means that 22 new potential antigens were selected at this step.

### 3.2. Protein Selection according to Antigenicity and Number of B-Epitopes

To refine the selection, the 22 preselected proteins were submitted to the VaxiJen server for antigenicity prediction. Antigenicity scores ranged from 0.4511 to 0.7827. This step allowed the elimination of two proteins with an antigenicity score lower than 0.5 (YP_001000503.1 and YP_001000297.1) (Table 1). The VaxiJen software indicated that all other candidates were antigenic (score > 0.5).

Each antigenic protein was assessed in terms of B-epitopes using BCPreds and AAP algorithms, and each B-epitope was studied for its antigenicity. Table 1 summarizes the number of B-epitopes predicted for each protein and each algorithm as well as the ratio between the number of B-epitopes and protein length.

### 3.3. Conservation of the Selected Proteins in the Sequenced* C. jejuni* and* C. coli* Strains

tblastn analyses were performed in order to assess the individual sharing of the preselected proteins among* C. jejuni* and* C. coli* strains. As shown in Table 2, all the proteins were shared with available* C. coli* strains except YP_001001027.1, which was also poorly shared with the available* C. jejuni* strains (6%). This protein was therefore removed from the list of potential vaccine antigens. Of the remaining 19 shortlisted proteins, five—YP_001001371.1, YP_001000248.1, YP_001000204.1, YP_001000654.1 and YP_001000615.1—were removed from the candidate list because of poor sharing among* C. jejuni* strains (<80%).

Table 2 also shows that none of the proteins are expressed by* Gallus gallus*.

### 3.4. Final Selection

Table 3 shows the final selection of potential vaccine candidates after the whole bioinformatic analysis process. Fourteen candidates were selected. Of these, three are extracellular proteins whereas the others are outer membrane proteins. Four flagellar proteins were identified and several were not characterized and designated as hypothetical proteins.

## 4. Discussion

In the last decades, advances in genomics, genome sequencing, and annotation, coupled with the development of bioinformatic tools has revolutionized vaccine development strategy. Reverse vaccinology allows vaccines to be designed even for noncultivable pathogens; genome availability is the only factor enabling* in silico* analysis or not. All the proteins are targeted even if only transiently expressed or scarce during infection. Furthermore, this strategy considerably reduces the time needed to develop new vaccines [35]. Reverse vaccinology was first successfully applied to the development of a vaccine against B serogroup* Neisseria meningitidis* [36]. Despite available prophylactic vaccines based on capsular polysaccharides (CPS) for four* N. meningitidis* serogroups (A, C, W, and Y), the development of a capsular vaccine against serogroup B was not possible because of CPS mimicry of polysialic acid in human cells.* In silico* analysis identified three proteins (fHbp, NadA, and NHBA) which were combined with outer membrane vesicles containing known antigen PorA and led to the European licensure of the 4CMenB vaccine in 2013 [37]. This strategy was then applied to several other pathogens such as herpes simplex viruses using the Vaxign program [38],* Staphylococcus aureus* for the* in silico* characterization of ten surface-exposed proteins [26],* Mycobacterium tuberculosis* with the identification of six novel antigen candidates to improve the tuberculosis vaccine [39], or* Streptococcus pneumonia* with the bioinformatic assessment of 13 protein targets [40]. The antigenicity and efficiency of the potential candidates selected in these last* in silico* studies have not yet been tested* in vitro* or* in vivo*.

Until now, and despite many studies, conventional development of a vaccine against* Campylobacter* in poultry has not led to an efficient vaccine in terms of immunogenicity and protection. Since 2005,* Campylobacter* has been and remains today the leading cause of bacterial foodborne gastroenteritis in Europe [1]. As poultry vaccination is one of the potential ways of reducing the incidence of human campylobacteriosis, it is important to pursue efforts to test new vaccine antigens. Reverse vaccinology is a suitable strategy to this end.

This* in silico* study predictively identified new vaccine antigens against* Campylobacter*. The reference* C. jejuni* ATCC (American Type Culture Collection) strain 81-176 was chosen for antigen prediction because of its high virulence in human diseases (namely, the chicken vaccine strategy is to prevent human infections). Even if this strain is not a good colonizer for chickens, this strain has been successfully used in several poultry experiments with high colonization levels [18]. Moreover, the reverse vaccinology aims to identify shared proteins among many* Campylobacter* strains (here more than 100 strains). Thereby, other more avian colonizer* Campylobacter* strains should be used for* in vivo* challenge experiments to evaluate the effectiveness of the proteins found by the bioinformatics analysis of the* C. jejuni* 81-176 genome.

Based on their cellular localization, adhesin-like properties, antigenicity, B-epitope density, and conservation among* Campylobacter* strains, 14 proteins were selected. It was decided to eliminate proteins with a sharing percentage lower than the flagellin sharing percentage. It has already been observed that flagellin could not be used as a vaccine candidate because of poor sharing among* Campylobacter* strains and the lack of cross-protection [14]. The known vaccine antigens of flagellins A and B were also identified alongside potential antigens using the same criteria. This strengthens the validity of the bioinformatic protocol used, because the flagellin has already been described and used as the immune-dominant antigen of* Campylobacter* [9, 11, 12]. However, it is important to keep in mind that the identified proteins were selected on the basis of predictions by various algorithms. Only* in vitro* and more* in vivo* experiments will confirm or refute the proteins' immune power. In terms of antigen ranking, proteins with a high antigenicity score and B-epitope density seem to be the best vaccine candidates and should therefore be evaluated for* in vivo* immunogenicity as a priority. Indeed, it has already been demonstrated that a high epitope density significantly enhances antigenicity and immunogenicity [41]. This strategy, being based on genome analysis, does not take into consideration lipid and saccharide antigens, which could also have immune properties. Concerning* Campylobacter*, capsule polysaccharides are not targeted through the reverse vaccinology protocol, although they could be immunogenic [42].

Several of the identified proteins had already been characterized and were mainly associated with* Campylobacter* virulence [43]. This is the case for three selected flagellar proteins—FlgE-1, FlgK, and FlgH—involved in* Campylobacter* motility, essential for bacteria survival in the gastrointestinal tract. These proteins were recently tested* in vitro* along with other flagellar proteins [44]. The first two were immunostained by more than 70% of tested sera from chickens older than 5-6 weeks; the third one was immunostained by 50% of the tested sera. The present* in silico* analysis is in line with these* in vitro* results, leading us to consider these three flagellar proteins as a potential vaccine antigen. However, no* in vivo* assessment is available yet. The FliD flagellar protein was similarly tested* in vitro* and was observed to react strongly to sera from chickens over 4 weeks of age [45]. In the present analysis, this flagellar protein was eliminated from the shortlist because of poor sharing with other* Campylobacter* strains (41%). Moreover, the ChuA protein—involved in the iron uptake system—had already been tested in an avian vaccine experiment using attenuated* Salmonella* as a vector [15] and did not significantly reduce cecal* Campylobacter* counts. Furthermore, the major PorA outer membrane protein was tested* in vivo* in a mouse model [46]. Mice vaccinations led to significantly higher antibody levels in serum and intestinal lavage fluids. A decrease in* C. jejuni* colonization levels was also observed after a heterologous challenge. Phospholipase A (PldA) and lipoprotein JlpA are involved in* Campylobacter* adhesion since it has been demonstrated that mutations of* pldA* impair the ability of* C. jejuni* to colonize cecum [47] and since Jin et al. highlighted the interaction of JlpA with a surface-exposed protein of epithelial cells [48].

To conclude, reverse vaccinology—a powerful tool for identifying new vaccine antigens—allowed 14 candidates to be selected for the development of a vaccine against* Campylobacter* in poultry. Several antigens identified as potential vaccine candidates are currently under* in vitro* and* in vivo* investigations to evaluate their immunogenicities and protective potentials against* Campylobacter* in chickens.

## Figures and Tables

**Table 1:** Potential vaccine candidates selected by the Vaxign program. Localization and length were obtained by the Vaxign program, antigenic score was obtained by the VaxiJen program, and the number of B-cell epitopes was obtained from both BCPreds and AAP methods. The calculated ratio between the number of B-epitopes and protein length is also shown. The two candidates eliminated because of a low antigenic score are shown in italics.

Protein accession	Description	ID	Localization	Length (aa)	Vaxijen score	BCPreds B-epitopes		AAP B-epitopes	
						*N*	Ratio *N*/length	*N*	Ratio *N*/length
YP_001000996.1	Flagellin B	FlaB	Extracellular	576	0.7650	10	0.017	11	0.019
YP_001000997.1	Flagellin A	FlaA	Extracellular	576	0.8185	11	0.019	12	0.021

YP_001001371.1	Flagellar hook protein	FlgE	Extracellular	838	0.7659	16	0.019	18	0.021
YP_001000562.1	Flagellin protein family		Extracellular	750	0.6965	15	0.020	15	0.020
YP_001000248.1	Flagellar capping protein	FliD	Extracellular	642	0.7021	11	0.017	11	0.017
YP_001000204.1	Putative periplasmic protein		OMP	553	0.6702	11	0.021	11	0.021
YP_001000654.1	Putative periplasmic protein		OMP	553	0.6702	11	0.021	11	0.021
YP_999769.1	Flagellar hook protein	FlgE-1	Extracellular	545	0.6567	10	0.018	12	0.022
YP_001001115.1	Flagellar hook-associated protein	FlgK	Extracellular	608	0.5836	11	0.018	10	0.016
YP_001000153.1	TonB-dependent receptor, putative, degenerate		OMP	704	0.5437	12	0.017	9	0.013
YP_001000945.1	N-Acetylmuramoyl-L-alanine amidase		OMP	659	0.6475	9	0.014	11	0.017
YP_001001027.1	Serine protease		OMP	1121	0.5268	9	0.008	11	0.010
YP_001000437.1	Putative OMP		OMP	508	0.6122	9	0.018	6	0.012
YP_999838.1	Hypothetical protein		OMP	400	0.6809	5	0.013	9	0.023
YP_999817.1	Hypothetical protein		OMP	315	0.7827	6	0.019	7	0.022
YP_001000383.1	Flagellar basal body L-ring protein	FlgH	OMP	232	0.6978	6	0.026	4	0.017
YP_001000935.1	Major OMP	PorA	OMP	424	0.6051	5	0.012	5	0.012
YP_001001008.1	Phospholipase A	PldA	OMP	329	0.5819	4	0.012	3	0.009
YP_001001257.1	TonB-dependent heme receptor	ChuA	OMP	702	0.6213	7	0.010	5	0.007
YP_001000615.1	Hypothetical protein		Extracellular	294	0.5498	3	0.010	3	0.010
YP_001000663.1	Surface-exposed lipoprotein	JlpA	OMP	372	0.6642	2	0.005	3	0.008
YP_001000261.1	Hypothetical protein		OMP	309	0.5149	2	0.006	3	0.010
*YP_001000503.1*	*Hypothetical protein*		*Extracellular*	*444*	*0.4603*	*/*	*/*	*/*	*/*
*YP_001000297.1*	*Major antigenic peptide*	*PEB4*	*OMP*	*273*	*0.4511*	*/*	*/*	*/*	*/*

**Table 2:** Potential vaccine candidates selected after blast analysis. tblastn analyses were performed for each amino acid sequence against both *C. jejuni* and *C. coli* whole genomes available on the NCBI site. The amount and percentage of sharing among the available genomes were determined. A blastp analysis was also performed against the host *Gallus gallus*. The six candidates eliminated because of poor sharing among *Campylobacter* strains are shown in italics.

Protein accession	Description	ID	Sharing among *C. jejuni* strains		Sharing among *C. coli* strains		Similarity in *Gallus gallus*
			*N*/93	%	*N*/9	%	
YP_001000996.1	Flagellin B	FlaB	77	83	9	100	No
YP_001000997.1	Flagellin A	FlaA	75	81	9	100	No

*YP_001001371.1*	*Flagellar hook protein*	*FlgE*	*15*	*16*	*8*	*89*	*No*
YP_001000562.1	Flagellin protein family		93	100	9	100	No
*YP_001000248.1*	*Flagellar capping protein*	*FliD*	*38*	*41*	*9*	*100*	*No*
*YP_001000204.1*	*Putative periplasmic protein*		*2*	*2*	*9*	*100*	*No*
*YP_001000654.1*	*Putative periplasmic protein*		*2*	*2*	*9*	*100*	*No*
YP_999769.1	Flagellar hook protein	FlgE-1	93	100	9	100	No
YP_001001115.1	Flagellar hook-associated protein	FlgK	93	100	9	100	No
YP_001000153.1	TonB-dependent receptor, putative, degenerate		90	97	9	100	No
YP_001000945.1	N-Acetylmuramoyl-L-alanine amidase		93	100	9	100	No
*YP_001001027.1*	*Serine protease*	*PEB4*	*6*	*6*	*1*	*11*	*No*
YP_001000437.1	Putative OMP		89	96	6	67	No
YP_999838.1	Hypothetical protein		93	100	9	100	No
YP_999817.1	Hypothetical protein		92	99	9	100	No
YP_001000383.1	Flagellar basal body L-ring protein	FlgH	93	100	9	100	No
YP_001000935.1	Major OMP	PorA	81	87	9	100	No
YP_001001008.1	Phospholipase A	PldA	92	99	9	100	No
YP_001001257.1	TonB-dependent heme receptor	ChuA	93	100	9	100	No
*YP_001000615.1*	*Hypothetical protein*		*64*	*69*	*9*	*100*	*No*
YP_001000663.1	Surface-exposed lipoprotein	JlpA	93	100	9	100	No
YP_001000261.1	Hypothetical protein		92	99	9	100	No

**Table 3:** Potential vaccine candidates selected after the whole bioinformatic analysis process including Vaxign and VaxiJen programs, BCPreds and AAP algorithms, and blast analyses. Of 1758 ORFs encoded by *C. jejuni*, strain 81-176 genome, 14 proteins were selected as vaccine candidates.

Protein accession	Description	Localization	ID
YP_001000562.1	Flagellin protein family	Extracellular	
YP_999769.1	Flagellar hook protein	Extracellular	FlgE-1
YP_001001115.1	Flagellar hook-associated protein	Extracellular	FlgK
YP_001000153.1	TonB-dependent receptor, putative, degenerate	OMP	
YP_001000945.1	N-Acetylmuramoyl-L-alanine amidase	OMP	
YP_001000437.1	Putative OMP	OMP	
YP_999838.1	Hypothetical protein	OMP	
YP_999817.1	Hypothetical protein	OMP	
YP_001000383.1	Flagellar basal body L-ring protein	OMP	FlgH
YP_001000935.1	Major OMP	OMP	PorA
YP_001001008.1	Phospholipase A	OMP	PldA
YP_001001257.1	TonB-dependent heme receptor	OMP	ChuA
YP_001000663.1	Surface-exposed lipoprotein	OMP	JlpA
YP_001000261.1	Hypothetical protein	OMP	
